# Supplementary material for: Knowledge, awareness and attitude among practicing oral and maxillofacial surgeons towards tele-dentistry: A cross-sectional survey
Source: PLoS One. 2026 Apr 8;21(4):e0346591. doi: 10.1371/journal.pone.0346591 (PMC13061227; doi:10.1371/journal.pone.0346591)
Supplement: Attachment — Submitted filename: PONE-D-25-32213.pdf [file pone.0346591.s001.pdf]

PLOS ONE

Knowledge, Awareness and Attitude among Practicing Oral and Maxillofacial Surgeons on Tele-Dentistry: A Cross-Sectional Survey

--Manuscript Draft--

**Manuscript Number:**	PONE-D-25-32213
**Article Type:**	Research Article
**Full Title:**	Knowledge, Awareness and Attitude among Practicing Oral and Maxillofacial Surgeons on Tele-Dentistry: A Cross-Sectional Survey
**Short Title:**	Perception of tele-dentistry among oral and maxillofacial surgeons
**Corresponding Author:**	Ayse Ozcan Kucuk<br>Mersin University: Mersin Universitesi<br>Yenişehir/Mersin, TÜRKIYE
**Keywords:**	tele-dentistry, oral and maxillofacial surgeon, knowledge, survey
**Abstract:**	Tele-dentistry is a telemedicine practice that uses telecommunications and technology. The aim of this study is to evaluate the perceptions and practices of oral and maxillofacial surgeons on tele-dentistry.This survey-based cross-sectional study was conducted among oral and maxillofacial surgeons in Türkiye, who are research assistants, specialists, doctor faculty members, associate professors and professors. The questionnaire was sent by the the Association of Oral and Maxillofacial Surgery of Türkiye (AÇBİD) to members on an e-mail of approximately 900 members using Google Forms. The questionnaires included questions assessing knowledge, awareness, and attitudes towards tele-dentistry. Data were analyzed statistically and p< 0.05 was considered significant. A total of 152 oral and maxillofacial surgeons from Türkiye participated. While 46.1% had heard of tele-dentistry, only 9.9% had used it, and 42.1% believed it could be applied in oral and maxillofacial surgery. There was a statistically significant difference between hearing about tele-dentistry and using tele-dentistry (p<0.001). A statistically significant difference was found between having heard of tele-dentistry, using it, and considering using it in the future, and age and title (p<0.001). The probability of dentists hearing about and using tele-dentistry was significantly lower in the younger age group compared to the other age groups (p<0.001). As the title increased, the rate of hearing about and using tele-dentistry increased, and expectations of its use in the future also increased (p<0.05).This study indicates that oral and maxillofacial surgeons in Türkiye possess limited knowledge and awareness of tele-dentistry, and that this modality remains insufficiently integrated into clinical practice. The inadequacy may be due to factors such as limited professional experience, concerns about data security, and inadequate institutional training. More education and dissemination of information about the application areas and technical aspects of tele-dentistry may lead to greater adoption by oral and maxillofacial surgeons.
**Order of Authors:**	Ayse Ozcan Kucuk
	Ali Menteş
	Adalet Celebi
	Fethi Atıl
**Additional Information:**	
**Question**	**Response**
**Financial Disclosure**<br><br>Enter a financial disclosure statement that describes the sources of funding for the work included in this submission. Review the submission guidelines for detailed requirements. View published research	The author(s) received no specific funding for this work.

Powered by Editorial Manager® and ProduXion Manager® from Aries Systems Corporation

articles from *PLOS ONE* for specific examples.<br><br>This statement is required for submission and **will appear in the published article** if the submission is accepted. Please make sure it is accurate.<br><br>**Funded studies**<br>Enter a statement with the following details:<br>• Initials of the authors who received each award<br>• Grant numbers awarded to each author<br>• The full name of each funder<br>• URL of each funder website<br>• Did the sponsors or funders play any role in the study design, data collection and analysis, decision to publish, or preparation of the manuscript?<br><br>Did you receive funding for this work?	
**Competing Interests**<br><br>Use the instructions below to enter a competing interest statement for this submission. On behalf of all authors, disclose any competing interests that could be perceived to bias this work—acknowledging all financial support and any other relevant financial or non-financial competing interests.<br><br>This statement is required for submission and **will appear in the published article** if the submission is accepted. Please make sure it is accurate and that any funding sources listed in your Funding Information later in the submission form are also declared in your Financial Disclosure statement.<br><br>View published research articles from *PLOS ONE* for specific examples.	The authors have declared that no competing interests exist.

Powered by Editorial Manager® and ProduXion Manager® from Aries Systems Corporation

**NO authors have competing interests**<br><br>Enter: *The authors have declared that no competing interests exist.*<br><br>**Authors with competing interests**<br><br>Enter competing interest details beginning with this statement:<br><br>*I have read the journal's policy and the authors of this manuscript have the following competing interests: [insert competing interests here]*<br><br>* typeset	
**Ethics Statement**<br><br>Enter an ethics statement for this submission. This statement is required if the study involved:<br><br>• Human participants<br>• Human specimens or tissue<br>• Vertebrate animals or cephalopods<br>• Vertebrate embryos or tissues<br>• Field research<br><br>Write "N/A" if the submission does not require an ethics statement.<br><br>General guidance is provided below. Consult the submission guidelines for detailed instructions. **Make sure that all information entered here is included in the Methods section of the manuscript.**	Ethical approval was obtained from Clinical Research Ethics Committee of Mersin University for this study (Approval number:2023/834).

**Format for specific study types**<br><br>**Human Subject Research (involving human participants and/or tissue)**<br>• Give the name of the institutional review board or ethics committee that approved the study<br>• Include the approval number and/or a statement indicating approval of this research<br>• Indicate the form of consent obtained (written/oral) or the reason that consent was not obtained (e.g. the data were analyzed anonymously)<br><br>**Animal Research (involving vertebrate animals, embryos or tissues)**<br>• Provide the name of the Institutional Animal Care and Use Committee (IACUC) or other relevant ethics board that reviewed the study protocol, and indicate whether they approved this research or granted a formal waiver of ethical approval<br>• Include an approval number if one was obtained<br>• If the study involved *non-human primates*, add *additional details* about animal welfare and steps taken to ameliorate suffering<br>• If anesthesia, euthanasia, or any kind of animal sacrifice is part of the study, include briefly which substances and/or methods were applied<br><br>**Field Research**<br><br>Include the following details if this study involves the collection of plant, animal, or other materials from a natural setting:<br>• Field permit number<br>• Name of the institution or relevant body that granted permission	
**Data Availability**<br><br>Authors are required to make all data underlying the findings described fully available, without restriction, and from the time of publication. PLOS allows rare exceptions to address legal and ethical concerns. See the PLOS Data Policy and FAQ for detailed information.	Yes - all data are fully available without restriction

A Data Availability Statement describing where the data can be found is required at submission. Your answers to this question constitute the Data Availability Statement and **will be published in the article**, if accepted.<br><br>**Important:** Stating 'data available on request from the author' is not sufficient. If your data are only available upon request, select 'No' for the first question and explain your exceptional situation in the text box.<br><br>Do the authors confirm that all data underlying the findings described in their manuscript are fully available without restriction?	
**Describe where the data may be found in full sentences. If you are copying our sample text, replace any instances of XXX with the appropriate details.**<br><br>• If the data are **held or will be held in a public repository**, include URLs, accession numbers or DOIs. If this information will only be available after acceptance, indicate this by ticking the box below. For example: *All XXX files are available from the XXX database (accession number(s) XXX, XXX.).*<br>• If the data are all contained **within the manuscript and/or Supporting Information files**, enter the following: *All relevant data are within the manuscript and its Supporting Information files.*<br>• If neither of these applies but you are able to provide **details of access elsewhere**, with or without limitations, please do so. For example:<br><br>*Data cannot be shared publicly because of [XXX]. Data are available from the XXX Institutional Data Access / Ethics Committee (contact via XXX) for researchers who meet the criteria for access to confidential data.*<br><br>*The data underlying the results presented in the study are available from (include the name of the third party*	The data underlying the results presented in the study are available from (Associate Professor Dr. Ayse OZCAN KUCUK E-mail address: ayseozcan89@hotmail.com Phone number: +90 324 361 00 01).

and contact information or URL).<br>• This text is appropriate if the data are owned by a third party and authors do not have permission to share the data.<br><br>* typeset	
Additional data availability information:	Tick here if your circumstances are not covered by the questions above and you need the journal's help to make your data available.

Revised Manuscript File Click here to access/download;Manuscript;Manuscript File (1).docx

Knowledge, awareness and attitude among practicing oral and maxillofacial surgeons on tele-dentistry: A Cross-Sectional survey

Ayse Ozcan-Kucuk, DDS, MDS[1,*], Ali Mentes, DDS[1], Adalet Celebi, DDS, MDS[1], Fethi Atıl, DDS, PhD[1]

[1] Department of Oral and Maxillofacial Surgery, Faculty of Dentistry, Mersin University, Mersin, Türkiye

Ayse Ozcan Kucuk

Associate Professor, Department of Oral and Maxillofacial Surgery, Faculty of Dentistry, Mersin University, 33343, Mersin, Türkiye

E-mail address: ayseozcan89@hotmail.com

Ali Mentes

Research Assistant, Department of Oral and Maxillofacial Surgery, Faculty of Dentistry, Mersin University, 33343, Mersin, Türkiye

E-mail address: dtalimentes@gmail.com

Adalet Celebi

Associate Professor, Department of Oral and Maxillofacial Surgery, Faculty of Dentistry, Mersin University, 33343, Mersin, Türkiye

E-mail address: adalet_celebi@hotmail.com

Fethi Atıl

Professor, Department of Oral and Maxillofacial Surgery, Faculty of Dentistry, Mersin University, 33343, Mersin, Türkiye

E-mail address: atil.fethi@gmail.com

[*]Corresponding author

Ayse Ozcan Kucuk

Full postal address: Mersin University, Faculty of Dentistry, Department of Oral and Maxillofacial Surgery, Mersin, 33343, Türkiye

E-mail address: ayseozcan89@hotmail.com

Phone number: +90 324 361 00 01

Fax number: +90 324 361 03 69

Knowledge, awareness and attitude among practicing oral and maxillofacial surgeons on tele-dentistry: A cross-sectional survey

Abstract

Tele-dentistry is a telemedicine practice that uses telecommunications and technology. The aim of this study is to evaluate the perceptions and practices of oral and maxillofacial surgeons on tele-dentistry.This survey-based cross-sectional study was conducted among oral and maxillofacial surgeons in Türkiye, who are research assistants, specialists, doctor faculty members, associate professors and professors. The questionnaire was sent by the the Association of Oral and Maxillofacial Surgery of Türkiye (AÇBİD) to members on an e-mail of approximately 900 members using Google Forms. The questionnaires included questions assessing knowledge, awareness, and attitudes towards tele-dentistry. Data were analyzed statistically and $p < 0.05$ was considered significant. A total of 152 oral and maxillofacial surgeons from Türkiye participated. While 46.1% had heard of tele-dentistry, only 9.9% had used it, and 42.1% believed it could be applied in oral and maxillofacial surgery. There was a statistically significant difference between hearing about tele-dentistry and using tele-dentistry ($p<0.001$). A statistically significant difference was found between having heard of tele-dentistry, using it, and considering using it in the future, and age and title ($p<0.001$). The probability of dentists hearing about and using tele-dentistry was significantly lower in the younger age group compared to the other age groups ($p<0.001$). As the title increased, the rate of hearing about and using tele-dentistry increased, and expectations of its use in the future also increased ($p<0.05$).This study indicates that oral and maxillofacial surgeons in Türkiye possess limited knowledge and awareness of tele-dentistry, and that this modality remains insufficiently

integrated into clinical practice. The inadequacy may be due to factors such as limited professional experience, concerns about data security, and inadequate institutional training. More education and dissemination of information about the application areas and technical aspects of tele-dentistry may lead to greater adoption by oral and maxillofacial surgeons.

Keywords: tele-dentistry, oral and maxillofacial surgeon, knowledge, survey

Introduction

Telemedicine is defined as “healthcare services provided using information and communication technologies for the diagnosis, treatment, and prevention of diseases, as well as research, training of healthcare workers, and improving public health, in situations where distance is important” in the report published by the World Health Organization (WHO) [1]. With the rapid development of technology, innovative practices such as telemedicine have become widespread in healthcare services. One of these innovative practices is tele-dentistry, which uses telecommunication and technology in processes such as consultation, diagnosis, treatment, and education to raise public awareness about oral and dental health [2].

Tele-dentistry provides significant convenience for both healthcare professionals and patients. Thanks to remote access, patients receive faster treatment, the burden on healthcare professionals is reduced, and patient waiting times are shortened. Moreover, bedbound patients and individuals at risk of infection can receive treatment more safely. Thanks to these advantages, society adopts remote healthcare services more easily [3]. However, in order for these services to be integrated into a country's health system and become widespread, it is essential that society shows positive acceptance of these services. In this context, determining the level of knowledge, attitudes, skills, and opinions of the society on remote healthcare

services is of great importance. Additionally, various studies should be carried out to raise awareness in the society and to ensure the adoption of these services [4].

As an important part of public health, dental professionals play a critical role in the delivery of remote healthcare services. Therefore, determining their views on telemedicine practices is important to increase the effectiveness of the services. The high level of knowledge and awareness of dentists about telemedicine is one of the basic elements that will ensure the widespread use of these practices. Moreover, their positive attitude towards telemedicine practices will contribute to the faster and more efficient delivery of healthcare services. Successful integration of this innovative approach to public health into the system is possible by continuously training and informing healthcare professionals about telemedicine. Despite the high burden of oral, dental and maxillofacial diseases in Türkiye, there are still no necessary foundations for tele-dentistry, as a critical component for public access to care, and establishment in the oral healthcare system. To create the capacity and necessary infrastructures for using tele-dentistry in the healthcare system, especially in developing countries such as Türkiye, it is critical to improve the awareness, attitude and knowledge of oral and maxillofacial surgeons about tele-dentistry, its benefits, drawbacks, and usage in practice [5]. Although there are studies in various countries evaluating the knowledge, attitudes, and awareness of dentists, dental students, and orthodontists regarding tele-dentistry [5-11], there is no study in the literature evaluating the knowledge, attitude and awareness of oral and maxillofacial surgeons on tele-dentistry [12]. In the literature, there is only one study examining the use of telemedicine in oral and maxillofacial surgery. This single study was conducted to determine the perceived utility and demand for application of telemedicine for improved patient care between 226 non-surgical dental practitioners and 41 oral and maxillofacial surgeons in Virginia [12]. However, there is a lack of previous literature on the perception and awareness of oral surgeons in Türkiye regarding tele-dentistry and its use in the delivery of health services.

Therefore, this study aims to evaluate the perceptions and practices of oral and maxillofacial surgeons in Türkiye regarding tele-dentistry.

Methods

Study Design and Population

This is a cross-sectional study based on a questionnaire conducted between January 3, 2024 and September 30, 2024. The data were collected from January 3, 2024 to March 31, 2024. The study population consisted of 152 oral and maxillofacial surgeons in Türkiye.

Ethical Approval

Ethical approval was obtained from Clinical Research Ethics Committee of Mersin University for this study (2023/834). The participants in this study were provided with detailed information about the study through the information text at the beginning of the questionnaire, and online informed consent was obtained from each participant electronically via Google Forms (Google LLC, Mountain View, CA, USA).

Data collection

For this study, the questionnaire developed by George et al. [13] was modified and used. The modified questionnaire consisting of 26 closed-ended questions was sent by the Association of Oral and Maxillofacial Surgery of Türkiye (AÇBİD) on an email list of approximately 900 members using Google Forms (Google LLC, Mountain View, CA, USA). The first part of the questionnaire included socio-demographic information, and the second part included questions on knowledge, awareness, and attitudes regarding tele-dentistry. Prior to roll-out, a pilot study was conducted on 8 oral and maxillofacial surgeons and changes were made for comprehension, syntax, and clarity.

Inclusion and Exclusion Criteria

The inclusion criteria for this study were as follows:

- Participants who participated in the study between January 2024 and March 2024
- Participants who were aged 23 and over
- Oral and maxillofacial surgeon professionals who agreed to participate in the study (Specialist, Assistant Professor, Associate Professor, Professor)
- Dentists who were continuing their specialization or doctoral training in oral and maxillofacial surgeon and who agreed to participate in the study (Research Assistant)

The exclusion criteria for this study were as follows:

- Specialization branches other than oral and maxillofacial surgeon
- General dental practitioners
- Participants included in the study outside January 2024 and March 2024
- Those who did not agree to participate in the study

Questionnaire Distribution

The quantitative portion of the study utilized convenience sampling. the Associati[illegible]f Oral and Maxillofacial Surgery (AÇBİD), a representative organization for oral and maxillofacial surgeons in Türkiye, was asked to distribute the questionnaire among its members via email. A total of 6 reminders were sent to the participants who did not respond every 2 weeks to ensure completion of the study. The questionnaire was open for a total period of 3 months. The questionnaire was sent to research assistants, specialist dentists, assistant professors, associate professors, and professors working in the field of oral and maxillofacial surgery. In total, 152 oral surgeons responded to the questionnaire. The non-response rate is unknown. Participation was entirely anonymous, and no personally identifiable information was collected at any stage. Participants had the right to change their responses or voluntarily withdraw from the questionnaire at any point without any consequences until submission. No incentive was offered for completing the questionnaire.

Statistical analysis

The study data were analyzed using the IBM SPSS V23 (SPSS Inc., Chicago, IL., USA) program. While the Pearson Chi-Square Test was used to examine the categorical data, the Bonferroni Correction Z Test was used to analyzed multiple comparisons. The analysis results were presented as frequency (percentage). The significance level was taken as $p<0.05$.

Results

This study included 152 responses from oral and maxillofacial surgeon in all regions of the country. Of the 152 participants consisting of dentists working in the field of oral and maxillofacial surgeon in Türkiye, 59.9% were male and 40.1% were female. 73.7% of the participants were between the ages of 23-35, and 46.1% had the title of research assistant. The demographic data of the participants are given in Table 1.

Table 1. Frequency distributions and descriptive statistics of the participants' characteristics.

**Variable**	**N**	**%**
Age		
23-35	112	73.7
35-45	30	19.7
45-55	9	5.9
>55	1	0.7
Gender		
Male	91	59.9
Female	61	40.1
Title		
Research assistant	70	46.1
Specialist	31	20.4
Assistant professor	20	13.1
Associate professor	19	12.5
Professor	12	7.9

According to the results of the questionnaire, 46.1% of the participants had heard of tele-dentistry before and 9.9% had used this service. The rate of those who thought that tele-dentistry was applicable in oral and maxillofacial surgeon was 42.1%, and the rate of participants who planned to use this service in the future was 49.7%. The responses of oral and maxillofacial surgeons regarding their knowledge, awareness, and attitudes towards tele-dentistry are presented in Table 2.

Table 2. The responses of oral and maxillofacial surgeons' knowledge, practice, attitude to use tele-dentistry			
**Questions**	**Yes**	**No**	**Neutral**
Have you heard of tele-dentistry?	70(46.1)	76(50)	6(3.9)
Have you used tele-dentistry before?	15(9.9)	135(89.4)	1(0.7)
Does tele-dentistry facilitate access to oral health services during pandemics?	84(55.3)	10(6.6)	58(38.1)
Can tele-dentistry be used in oral and maxillofacial surgery?	64(42.1)	25(16.5)	63(41.4)
Does tele-dentistry relate to the practice of using computers, internet and technologies to diagnose and advise on treatment remotely?	103(67.8)	2(1.3)	47(30.9)
Does tele-dentistry help the patient with consultation and diagnosis in oral and maxillofacial surgery during COVID?	105(69.1)	9(5.9)	38(25)
Does tele-dentistry help the patient to share radiographs for diagnosis in oral and maxillofacial surgery?	106(70.2)	4(2.6)	41(27.2)
Can tele-dentistry be used in the early detection, diagnosis and treatment of oral diseases?	95(62.5)	13(8.6)	44(28.9)
Can tele-dentistry be used in the diagnosis and treatment of patients with temporomandibular disorders?	64(42.1)	42(27.6)	46(30.3)
Can the knowledge and experience of other maxillofacial surgeons be utilized during the operation by remote connection in the operations of	84(55.6)	20(13.3)	47(31.1)
Can implant planning be done using tele-dentistry?	72(47.7)	36(23.8)	43(28.5)
Can tele-dentistry be used in the diagnosis and treatment planning of patients with maxillofacial trauma?	67(44.1)	41(27)	44(28.9)
Do you think tele-dentistry can be used in patient follow-up after minor oral surgical procedures such as impacted wisdom teeth extraction?	107(70.4)	14(9.2)	31(20.4)
Can tele-dentistry be used in the follow-up of MRONJ patients?	85(55.9)	31(20.4)	36(23.7)
Can maxillofacial surgeons evaluate patients in virtual meetings with other branches such as ear nose and throat (ENT) specialist and plastic	122(80.8)	3(2)	26(17.2)
Does tele-dentistry help non-specialist dentists to refer their patients to specialist dentists?	115(75.7)	2(1.3)	35(23)
Can tele-dentistry be used for training in dentistry and maxillofacial surgery?	94(61.8)	20(13.2)	38(25)
Does tele-dentistry help reduce clinical costs in dentistry?	84(55.3)	14(9.2)	54(35.5)
Do you think tele-dentistry saves time for dentists and maxillofacial surgeons?	81(53.3)	23(15.1)	48(31.6)
Does tele-dentistry facilitate access to patients in underserved areas (e.g. rural areas)?	98(64.5)	13(8.5)	41(27)
Can tele-dentistry be used for patient appointment scheduling?	103(68.2)	6(4)	42(27.8)
Does tele-dentistry violate patients' personal data and privacy?	29(19.1)	53(34.9)	70(46)
Can tele-dentistry be added to routine dental practice?	80(52.6)	20(13.2)	52(34.2)
Can tele-dentistry be used in other branches of dentistry other than oral and maxillofacial surgery?	111(73.5)	3(2)	37(24.5)
Do you think that examinations using computers, telephones and telecommunication technologies, as in the traditional clinical setting, are	45(30.2)	55(36.9)	49(32.9)
In the future, will you practice tele-dentistry?	75(49.7)	18(11.9)	58(38.4)

Notes: Data are presented as N and %

185

Among individuals who had heard of tele-dentistry, the rate of those who used tele-dentistry before was 21.7%, while the rate of those who did not use it was 78.3%. Additionally, a statistically significant relationship was found between the status of hearing about tele-dentistry and previous use (p<0.001) (Table 3).

Table 3. The relationship between hearing about tele-dentistry and previous use of tele-dentistry

	Have you heard of tele-dentistry?			Test statistics	P-value
	Yes	No	Neutral		
Have you used tele-dentistry before?					
Yes	15 (21.7) ^{a}	0 (0)^{b}	0 (0) ^{ab}		
No	54 (78.3) ^{a}	76(100) ^{b}	5 (83.3) ^{a}	43.93	**<0.001**
Neutral	0 (0)^{a}	0 (0)^{a}	1 (16.7) ^{b}		

Data are presented as N and % bold: p<0.05

Pearson Chi-Square Test; a-b: There is no difference between groups with the same letter

A statistically significant relationship was found between age and the status of hearing about tele-dentistry and previous use (p<0.001). Individuals in the younger age group are less likely to have heard of tele-dentistry than individuals in the middle age group (Table 4). The rate of using tele-dentistry among participants in the younger age group was found to be significantly lower than the rate of using tele-dentistry among participants aged 45 and over.

Table 4. The relationship between age and hearing about tele-dentistry, using it before and using it in the future

	Age			Test statistics	p-value
	23-35	35-45	45 and over		
Have you heard of tele-dentistry?					
Yes	42 (37.5) ^{a}	22(73.3) ^{b}	6 (60) ^{ab}		
No	66 (58.9) ^{a}	8 (26.7) ^{b}	2 (20) ^{ab}	21.654	**<0.001**
Neutral	4 (3.6) ^{ab}	0 (0)^{b}	2 (20)^{a}		
Have you used tele-dentistry before?					
Yes	9 (8)^{a}	3 (10) ^{ab}	3 (33.3) ^{b}	22.381	**<0.001**

No	103 (92)^{a}	27 (90)^{ab}	5 (55.6)^{b}		
Neutral	0 (0)^{a}	0 (0)^{ab}	1 (11.1)^{b}		
In the future, will you practice tele-dentistry?					
Yes	48 (42.9)^{a}	23 (79.3)^{b}	4 (40)^{ab}		
No	12 (10.7)^{a}	2 (6.9)^{a}	4 (40)^{b}	21.062	**<0.001**
Neutral	52 (46.4)^{a}	4 (13.8)^{b}	2 (20)^{ab}		

Data are presented as N and % bold: $p<0.05$

Pearson Chi-Square Test; a-b: There is no difference between groups with the same letter

A significant relationship was found between academic titles and the status of hearing about tele-dentistry, using it, and using it in the future (p=0.04, p=0.02, p=0.01, respectively) (Table 5). As the title rises, the rate of hearing about and using tele-dentistry increases, and expectations of its use in the future also increase. In particular, professors and associate professors' knowledge and usage rates of tele-dentistry were significantly higher than those of research assistants and specialists.

Table 5. The relationship between title and hearing about tele-dentistry, using it before and using it in the future

	Title					Test statistics	P-value
	Research assistant	Specialist	Assistant professor	Associate professor	Professor		
Have you heard of tele-dentistry?							
Yes	22 (31.4)^{a}	12(38.7)^{ab}	14 (70)^{b}	13 (68.4)^{b}	9 (75)^{b}		
No	46 (65.7)^{a}	17(54.8)^{ab}	6 (30)^{b}	5 (26.3)^{b}	2 (16.7)^{b}	22.513	**0.004**
Neutral	2 (2,9)	2 (6,5)	0 (0)	1 (5,3)	1 (8,3)		
Have you used tele-dentistry before?							
Yes	5 (7,1)^{a}	2 (6,5)^{a}	1 (5)^{ab}	2 (10.5)^{ab}	5 (45.5)^{b}		
No	65 (92,9)^{a}	29 (93,5)^{a}	19 (95)^{ab}	16 (84.2)^{ab}	6 (54.5)^{b}	24.118	**0.002**
Neutral	0 (0)	0 (0)	0 (0)	1 (5.3)	0 (0)		
In the future, will you practice tele-dentistry?							
Yes	28 (40)^{a}	12 (38.7)^{a}	11(57.9)^{ab}	17 (89.5)^{b}	7 (58.3)^{ab}		
No	6 (8.6)	7 (22.6)	1 (5.3)	1 (5.3)	3 (25)	25.331	**0.001**
Neutral	36 (51.4)^{a}	12 (38.7)^{ab}	7 (36.8)^{ab}	1 (5.3)^{b}	2 (16.7)^{ab}		

Data are presented as N and % bold: $p<0.05$

Pearson Chi-Square Test; a-b: There is no difference between groups with the same letter

A statistically significant relationship was found between the title and the status of previous use of tele-dentistry in participants aged 25-35 (p=0.002). The rates of using tele-dentistry among research assistants, specialists, assistant professors, and associate professors were found to be 7.1%, 4%, 7.1%, and 66.7%, respectively. It was observed that the rate of use of tele-dentistry by associate professors was significantly higher than the rate of use of tele-dentistry by research assistants and specialists (Table 6).

Table 6. The relationship between title and previous use of tele-dentistry among participants in the 25-35 age category

	Title				Test statistics	P-value
	Research assistant	Specialist	Assistant professor	Associate professor		
Have you used tele-dentistry before?						
Yes	5 (7.1) ^{a}	1 (4)^{a}	1 (7.1) ^{ab}	2 (66.7) ^{b}	14.597	**0.002**
No	65 (92.9)	24 (96)	13 (92.9)	1 (33.3)		

Data are presented as N and %   bold: p<0.05

Pearson Chi-Square Test; a-b: There is no difference between groups with the same letter

Discussion

Although telemedicine technology is being widely used in medicine and has reached in the initial application stages in dentistry and oral and maxillofacial surgery, there is no written study on its application and level of knowledge in oral and maxillofacial surgery in Türkiye. Therefore, the present study is the first to determine the knowledge, awareness, and attitudes of 152 oral and maxillofacial surgeons in Türkiye regarding tele-dentistry practices and to fill the knowledge gap in this area. In recent years, several studies have evaluated the knowledge, awareness, and attitudes of dentists, dentistry students and orthodontists about tele-dentistry in various countries [5,10,11,13-15]. Most of these studies have reported that dental practitioners

have poor knowledge and practical experience in tele-dentistry [8]. In the literature, there is a few studies examining the optimization, accuracy and use of telemedicine in oral and maxillofacial surgery [12,16-18]. A study by Wood et al. was conducted to determine the perceived utility and demand for application of telemedicine for improved patient care between non-surgical dental practitioners and oral and maxillofacial surgeons [12]. In the same study, the questionnaire was completed by 226 non-surgical dental practitioners and 41 oral and maxillofacial surgeons' respondents. Gangwani et al. conducted a study to determine the accuracy of planned oral and maxillofacial surgery procedures for patients seen initially by telemedicine in the department of oral and maxillofacial surgery during the pandemic [17] and this retrospective study sample consisted of patients, not oral and maxillofacial surgeons. The retrospective study by Miranda-Hoover et al. aimed to analyzed telehealth utilization for two specialty care practices: oral medicine and oral and maxillofacial surgery during the first 2 years of the pandemic, its impact as a new treatment modality and on participating providers, as well as identify the type of patient visit that most readily adopted telehealth [16]. This study reported that oral and maxillofacial surgery utilized telehealth 12% of the time, and oral medicine 8% of the time. Masongo et al. assessed the link between Australian oral and maxillofacial consultants' characteristics, perceptions, and willingness to sustain telehealth services and identified perceived barriers and unmet needs affecting their ongoing use of telehealth in oral and maxillofacial surgery [19]. In same study, a total of 42 oral and maxillofacial consultants of the 90 invited consultants participated in the study, yielding a response rate of 46.6%[19] and specifically, 65% agreed that reducing patient costs constitute notable advantages of telehealth. Masongo et al. revealed that most oral and maxillofacial surgeons perceive telehealth as beneficial for enhancing access to oral and maxillofacial healthcare, especially in rural and remote areas. In our study, approximately 55% of respondents believed that tele-dentistry would help reduce clinical costs in dentistry and save times for dentists and maxillofacial surgeons.

Additionally, approximately 65% of oral and maxillofacial surgeon believed that tele-dentistry facilitate access to patients in underserved areas.

The results of this study showed that 46.1% of oral and maxillofacial surgeons in Türkiye have knowledge about tele-dentistry, while their general knowledge and awareness levels are quite low. This situation indicates that awareness in the field of tele-dentistry should be increased. In some previous studies, similar to the findings in our study, it was found that dentists had a low level of knowledge about tele-dentistry, with rates ranging from 25.16% to 37.3% [5,14]. On the other hand, some studies reported that participants had a high level of knowledge, with rates ranging from 68.6% to 96.1% [10,11,13,15]. These conflicting results suggest that the prevalence and acceptance rates of tele-dentistry in Türkiye may vary depending on regional, educational, or occupational factors.

In this study, significant differences were found in terms of tele-dentistry awareness and experience among different age groups, suggesting that age has an impact on the levels of knowledge and awareness about tele-dentistry. The probability of dentists hearing about and using tele-dentistry was significantly lower in the younger age group compared to th[illegible] participants in the middle-aged and over 45 age groups ($p<0.001$). This suggests that younger dentists' interest in digital health technologies may be lower compared to that of their older colleagues. On the other hand, a study conducted in Kerala noted that younger age group participants had higher awareness about tele-dentistry. In the study in Kerala, it was found that 43.5% of the participants between the ages of 25-35 responded positively about tele-dentistry and there was a significant relationship between age group and knowledge ($p<0.032$) [13]. These contradictory results suggest that different geographical and cultural contexts may influence awareness levels regarding tele-dentistry. Low awareness among the young age group draws attention to the need for more efforts to disseminate training and digital health practices

in this field in Türkiye. The fact that Türkiye is a developing country may cause difficulties in accessing healthcare services and adopting digital technologies. The reasons for this situation may be that the health system has not been sufficiently integrated with these new digital practices, distrust of tele-health, privacy concerns, and cultural factors [11,20]. It is thought that middle-aged and older groups may be more open to technology and alternative solutions. Although young people are highly familiar with technology, their lack of knowledge about the practices of these technologies in the field of health prevents them from benefiting from healthcare services more effectively. This highlights the need for strategies to increase health literacy among youth in developing countries. Carrying out educational programs and information campaigns to disseminate tele-dentistry services will increase young people's knowledge about these services and strengthen their trust in the healthcare system. The inclusion of tele-dentistry services in dentistry specialization training will contribute to increasing awareness in this field and more effective adoption of the practice. Including tele-dentistry in dentistry specialization training will both enable young dentists to gain knowledge about digital health technologies and encourage them to use this technology more effectively in their clinical practices. Moreover, providing these trainings will help tele-dentistry to become more widely and effectively included in dental practice in the future [9,11,14,21,22].

The findings of our study revealed a significant relationship between academic title and knowledge, awareness, and use of tele-dentistry. It was observed that professors and associate professors, in particular, had more knowledge about tele-dentistry than research assistants and specialists, and their usage rates in this field were significantly higher. These findings are similar to the results of a study conducted in Saudi Arabia. This study reported that dentistry specialists and dentists with longer clinical experience had more knowledge and awareness about tele-dentistry and implemented more practices in this field. This shows that level of

education and clinical experience are determining factors in attitudes toward and use of innovative practices such as tele-dentistry [5].

However, the results of a study conducted in Pakistan reveal results that contradict the results of our study. In the relevant study conducted in Pakistan, it was stated that undergraduate dentists had higher awareness levels about tele-dentistry, but postgraduate students and trainees had lower awareness about the issue. This contradictory finding may be due to differences in the educational systems of both countries, duration of clinical experience, and cultural approaches to tele-dentistry practices. For example, while there may be a greater focus on tele-dentistry in undergraduate education in Pakistan, our study found that individuals with higher academic titles had more opportunities to follow and implement developments in this field. This study highlights that academic title and experience play an important role in the effective adoption of tele-dentistry practices; therefore, it reveals the importance of tailoring educational strategies in this field to local health and education systems [11,21].

The results of our study reveal the significant relationship between awareness and usage of tele-dentistry. Among individuals who had heard of tele-dentistry, the rate of those who had used this service before was 21.7%, while the rate of those who had not used it was 78.3%. Moreov[illegible] a statistically significant relationship was found between hearing about tele-dentistry and its usage ($p<0.001$). These findings show that having knowledge about tele-dentistry is an important factor in increasing the use of this service and that usage rates will increase as awareness increases. This result, which shows that knowledge and experience factors play a decisive role in accessing healthcare services, reveals that healthcare professionals and patients need to be more informed about the subject in order for tele-dentistry practices to become widespread. Furthermore, the results highlight the importance of education and awareness-raising programs on tele-dentistry and suggest that necessary steps should be taken to enable the health system to benefit more effectively from this new service [23].

Our study reveals that tele-dentistry plays an important role in healthcare and promotes collaboration between different specialties. In particular, the fact that the percentage of participants who thought that maxillofacial surgeons could evaluate patients by holding virtual meetings with other branches such as ear, nose, and throat and plastic surgery was 80.8% shows that tele-dentistry strengthens interdisciplinary approaches. This finding suggests that tele-dentistry offers healthcare professionals the opportunity to conduct patient management more comprehensively and effectively, while also helping healthcare professionals communicate more quickly and efficiently. Digital platforms create opportunities that enable healthcare professionals to provide higher quality services, not only through coordination in the treatment process, but also through information sharing and clinical decision support systems. Moreover, the fact that the percentage of participants who thought that non-specialist dentists helped refer their patients to specialist dentists was 75.7%, once again emphasizes the functionality and supporting role of tele-dentistry in the healthcare system. This collaboration not only ensures coordination among healthcare professionals, but can also increase the overall efficiency of the system by increasing patient access to more effective and timely healthcare services.

Our study provides important data regarding the acceptability and potential of tele-dentistry. The rate of those who think that tele-dentistry will violate personal data and privacy is 19.1%, which is a critical concern in terms of ensuring patient safety. Privacy and security issues in digital healthcare practices are frequently emphasized in literature, and many studies point out that security protocols for the protection of patient data need to be strengthened [24,25]. In this context, strengthening security measures may increase patients' confidence in tele-dentistry. On the other hand, the fact that the rate of those who questioned the accuracy of examinations performed with computer and telecommunication technologies in the traditional environment was 30.2% of indicates the existence of some doubts about the effectiveness of tele-dentistry. This finding is in line with some studies in the relevant literature where the necessity of physical

examination and the limitations of remote diagnosis are frequently discussed [26,27]. Despite the advantages it offers, such as accessibility and time savings, tele-dentistry also has some disadvantages and concerns. Therefore, it is important for future studies to focus more on patient safety and privacy issues to eliminate these disadvantages and increase the effectiveness of tele-dentistry practices.

Oral health professionals show growing interest in telehealth, particularly for remote patients [7]. A previous study observed that there is a willingness among Australian oral and maxillofacial consultants to continue using telehealth services in the future with 82% of the consultants from this study expressing their willingness to use it routinely [19]. In our study, 49.7% of the oral and maxillofacial surgeons stated that they were considering using tele-dentistry in the future. This result indicates an increasing interest in and potential use of tele-dentistry in Türkiye, where the tele-dentistry usage rate among oral and maxillofacial surgeons is 15%. There are similar findings in the literature that tele-dentistry has increased access to healthcare services and that rapid development has been made in this field, especially following the COVID-19 pandemic. The pressures of the pandemic on healthcare systems have accelerated the adoption of digital healthcare and reinforced the effectiveness of tele-dentistry [28]· Our study indicates that the use of tele-dentistry may increase in the future and that further research and development studies are needed in this area.

Limitations

The main limitations of this study were the relatively small sample size and the low response rate to the questionnaires. We managed to recruit 152 oral and maxillofacial surgeons, which, while statistically sufficient, may still limit the generalizability of our findings to the entire oral and maxillofacial surgeon community in Türkiye. However, an analysis of the demographic data indicates that we obtained a diverse range of participants from various age groups and professional backgrounds. This suggests that our sample can be considered reasonably

representative of the target population. Furthermore, our study was inspired by a previous study conducted by George et al. [13] in 2021, which included 150 participants. To the best of our knowledge, this is the first study to evaluate the perceptions and knowledge of oral surgeons in Türkiye regarding tele-dentistry and our sample size was similar to George et al. [13]. Nonetheless, our study adds to the existing literature and may serve as a foundation for future research with broader participation to validate and expand upon these findings.

Another limitation is related to the recruitment method. The participants were primarily recruited through AÇBİD, with the survey being disseminated online via Google Forms. Since participation was voluntary and conducted online, selection bias could have influenced the results. Individuals with greater interest in tele-dentistry may have been more inclined to participate, potentially leading to an overrepresentation of those who are more aware of or engaged with the topic. However, we believe that this potential bias does not significantly affect our findings, as the study included a broad demographic representation.

Furthermore, the study employed a convenience sampling method, which has inherent limitations regarding the generalizability of results. Ideally, a random sampling method would have been used to reduce selection bias. However, given the constraints of the study, it was not feasible to conduct a fully randomized recruitment process. Despite this, we believe that our findings provide valuable insights into the knowledge, awareness, and attitudes of oral and maxillofacial surgeon professionals toward tele-dentistry.

Lastly, as this was a cross-sectional study, the data represent a single point in time, preventing any conclusions about causality or trends over time. Future longitudinal studies could help assess how attitudes and awareness of tele-dentistry evolve among oral and maxillofacial surgeons. Another limitation of this study is that it did not consider patient perspectives; incorporating them into future studies could improve telehealth practice.

Despite these limitations, this study provides crucial baseline data on tele-dentistry awareness among oral and maxillofacial surgeons in Türkiye and highlights the need for further educational initiatives and policy development to enhance the integration of tele-dentistry into clinical practice.

Conclusions

Although the use of tele-dentistry is low among oral and maxillofacial surgeons in Türkiye, they view tele-dentistry positively and are willing to use tele-dentistry in the future. As a result of the study, it was determined that a significant portion of oral and maxillofacial surgeons were knowledgeable about tele-dentistry. However, it was observed that the rate of hearing about and using this service was lower than expected among young people. Additionally, it was found that the probability of adopting tele-dentistry increased as the academic title increased. Tele-dentistry has significant potential in the healthcare system, and information and education programs should be organized for wider acceptance and adoption among oral and maxillofacial surgeons in the future.

Abbreviations

WHO: World Health Organization; AÇBİD: Oral and Maxillofacial Surgery Society of Türkiye

Acknowledgements

Not applicable

Author contributions

Conceptualization: Ayşe Özcan Küçük, Ali Menteş, Adalet Çelebi, Fethi Atıl.

Data curation: Ayşe Özcan Küçük, Ali Menteş.

Formal analysis: Ayşe Özcan Küçük.

Funding acquisition: Ayşe Özcan Küçük, Ali Menteş, Adalet Çelebi, Fethi Atıl.

Investigation: Ayşe Özcan Küçük, Ali Menteş, Adalet Çelebi, Fethi Atıl.

Methodology: Ayşe Özcan Küçük.

Project administration: Ayşe Özcan Küçük, Ali Menteş, Adalet Çelebi.

Software: Ayşe Özcan Küçük.

Supervision: Ayşe Özcan Küçük.

Validation: Ayşe Özcan Küçük.

Visualization: Ayşe Özcan Küçük.

Writing – original draft: Ayşe Özcan Küçük, Ali Menteş, Adalet Çelebi, Fethi Atıl.

Writing – review & editing: Ayşe Özcan Küçük.

References

1. World Health Organization (WHO). Telemedicine: Opportunities and Developments in Member States. Report on the Second Global Survey on eHealth. Geneva, Switzerland: World Health Organization; 2010. Accessed January 12, 2024. https://iris.who.int/bitstream/handle/10665/44497/9789241564144_eng.pdf?sequence=1

2. Kirkwood B. Development of military teledentistry. *Med J (Ft Sam Houst Tex).* 2021;(PB 8-21-10/11/12):33-39. PMID: 34714920.

3. Islam MRR, Islam R, Ferdous S, et al. Teledentistry as an effective tool for communication improvement between dentists and patients: an overview. Healthcare (Basel) 2022;10(8):1586; doi: 10.3390/healthcare10081586. doi: 10.3390/healthcare10081586

4. Celep G, Budakoğlu İİ, Coşkun Ö. Teletıp ve tıp eğitiminde teletıp müfredatı. Tıp Eğitimi Dünyası 2024;23(70):5–23. doi: 10.25282/ted.1363407

5. Nassani MZ, Al-Maweri SA, AlSheddi A, et al. Teledentistry—knowledge, practice, and attitudes of dental practitioners in Saudi Arabia: a nationwide web-based survey. Healthcare (Basel) 2021;9(12):1682. doi: 10.3390/healthcare9121682

6. Flores-Mir C, Palmer NG, Northcott HC, et al. Perceptions and attitudes of Canadian dentists toward digital and electronic technologies. J Can Dent Assoc 2006;72(3):243. PMID: 16696889

7. Estai M, Kruger E, Tennant M. Perceptions of Australian dental practitioners about using telemedicine in dental practice. Br Dent J 2016;220(1):25–29. doi: 10.1038/sj.bdj.2016.25

8. Saberi Z, Rahimi A, Soheili L, et al. Knowledge and attitude of Iranian dental practitioners toward teledentistry: a web-based descriptive survey. Digit Health 2024;10:20552076241272590. doi: 10.1177/20552076241272590

9. Boringi M, Waghray S, Lavanya R, et al. Knowledge and awareness of teledentistry among dental professionals: a cross-sectional study. J Clin Diagn Res 2015;9(8):ZC41. doi: 10.7860/JCDR/2015/13303.6320

10. Pradhan D, Verma P, Sharma L, et al. Knowledge, awareness, and attitude regarding teledentistry among postgraduate dental students of Kanpur city, India: a questionnaire study. J Educ Health Promot 2019; 8:10. doi: 10.4103/jehp.jehp_363_18

11. Subhan R, Ismail WA, Musharraf S, et al. Teledentistry as a supportive tool for dentists in Pakistan. Biomed Res Int 2021; 2021:8757859. doi: 10.1155/2021/8757859

12. Wood EW, Strauss RA, Janus C, et al. The use of telemedicine in oral and maxillofacial surgery. J Oral Maxillofac Surg 2016;74(4):719–728. doi: 10.1016/j.joms.2015.11.026

13. George PP, Edathotty TT, Gopikrishnan S, et al. Knowledge, awareness, and attitude among practicing orthodontists on teledentistry during the COVID-19 pandemic in Kerala: a cross-sectional survey. J Pharm Bioallied Sci 2021;13(Suppl 1):846–850. doi: 10.4103/jpbs.JPBS_826_20

14. Aboalshamat KT. Awareness of, beliefs about, practices of, and barriers to teledentistry among dental students and the implications for Saudi Arabia Vision 2030 and coronavirus pandemic. J Int Soc Prev Community Dent 2020;10(4):431–437. doi: 10.4103/jispcd.JISPCD_183_2

15. Murererehe J, Uwambaye P, Isyagi M, et al. Knowledge, attitude, and practices of dental professionals in Rwanda towards the benefits and applications of teledentistry. Rwanda J 2017;4(1):39–47. doi: 10.4314/rj.v4i1.6F

16. Miranda-Hoover A, He P, Chau T, et al. Telehealth utilization in oral medicine and oral and maxillofacial surgery. Telemed J E Health 2024;30(3):780–787. doi: 10.1089/tmj.2023.0099

17. Gangwani P, Mooneyham R, Feng C, et al. Accuracy of telemedicine consultations in oral and maxillofacial surgery during the COVID-19 pandemic. J Oral Maxillofac Surg 2023;81(1):65–71; doi: 10.1016/j.joms.2022.09.016

18. Moon HS, Wang TT, Rajasekaran K, et al. Optimizing telemedicine encounters for oral and maxillofacial surgeons during the COVID-19 pandemic. Oral Surg Oral Med Oral Pathol Oral Radiol 2021;131(2):166–172. doi: 10.1016/j.oooo.2020.08.015

19. Masongo C, Daire J, Estai M, et al. Barriers and willingness to continue using telehealth services beyond the COVID-19 pandemic from the perspectives of oral and maxillofacial surgeons in Australia: a mixed-method study. Healthcare (Basel) 2024;12(20):2086. doi: 10.3390/healthcare12202086

20. Uluc Cİ, Ferman M. A comparative analysis of user insights for e-health development challenges in Turkey, Kingdom of Saudi Arabia, Egypt, and United Arab Emirates. J Manag Mark Logist 2016;3(2):176–189. doi: 10.17261/Pressacademia.2016219945

21. Alipour J, Farsadhabibi H, Karimi A. Knowledge and attitudes of dentistry students toward teledentistry. Appl Health Inf Technol 2020;1(1):11–18. doi: 10.18502/ahit.v1i1.5254

22. Daniel SJ, Kumar S. Teledentistry: a key component in access to care. J Evid Based Dent Pract 2014;14:201–208. doi: 10.1016/j.jebdp.2014.02.008

23. Patel RN, Antonarakis GS. Factors influencing the adoption and implementation of teledentistry in the UK, with a focus on orthodontics. Community Dent Oral Epidemiol 2013;41(5):424–431. doi: 10.1111/cdoe.12029

24. Alhammad N, Alajlani M, Abd-Alrazaq A, Epiphaniou G, Arvanitis T. Patients' perspectives on the data confidentiality, privacy, and security of mHealth apps: systematic review. J Med Internet Res. 2024;26:e50715. doi: 10.2196/50715

25. Stephens CD, Cook J. Attitudes of UK consultants to teledentistry as a means of providing orthodontic advice to dental practitioners and their patients. J Orthod 2002;29(2):137–142. doi: 10.1093/ortho/29.2.137

26. Gajarawala SN, Pelkowski JN. Telehealth benefits and barriers. J Nurse Pract. 2021;17(2):218–221. doi: 10.1016/j.nurpra.2020.09.013

27. King G, Richards H, Godden D. Adoption of telemedicine in Scottish remote and rural general practices: a qualitative study. J Telemed Telecare 2007;13(8):382–386. doi: 10.1258/135763307783064430

28. Jin-Yu ML, Min CW, Si Jin JL, Babar MG, Mahdi SS. Practical applications of teledentistry during the Covid-19 pandemic in ASEAN member states: a systematic review. BMC Oral Health. 2024;24(1):421. doi: 10.1186/s12903-024-04177-x
