# Supplementary material for: Knowledge, awareness and attitude among practicing oral and maxillofacial surgeons towards tele-dentistry: A cross-sectional survey
Source: PLoS One. 2026 Apr 8;21(4):e0346591. doi: 10.1371/journal.pone.0346591 (PMC13061227; doi:10.1371/journal.pone.0346591)
Supplement: Attachment — Submitted filename: report PONE-D-25.pdf [file pone.0346591.s002.pdf]

Reviewer report

Title: **Knowledge, Awareness and Attitude among Practicing Oral and Maxillofacial Surgeons on Tele-Dentistry: A Cross-Sectional Survey Overall assessment:**

Overall assessment

This is a valuable and timely piece of study and it does tackle an understudied area. If the study heightened the analysis and became more precise and clearer on the method the piece would be a meaningful addition to the literature on tele-dentistry use by oral and maxillofacial surgeons.

Abstract.

- There some abbreviations are not identified.

  The novelty (first survey among oral/maxillofacial surgeons in Türkiye) could be more prominently highlighted in the abstract. Although p-values are reported with precision, interpretability is limited because the effect sizes or the strength of associations are not stated.

1. Materials and methods

Sampling: convenience sampling method does raise some concerns regarding overall representation. The author should write how the sample size is selected? and calculated?

Questionnaire: While some parts of the study are based on previous research, there is no information on what changes were made, if any, and if their psychometric properties–validation and reliability, post-change–were determined, all these points should be considered and placed on the text.

Analysis. Only Chi-square and Bonferroni tests are referenced. I suggest to add regression analysis, which is very important to assess the independent predictors of awareness/usage more accurately.

Clarify how the non-response rate can affect generalizability.

2. Results/

2.1. The results are fair, they should cover all aspects of the study. The predictors such as age, title and independent variables can be reanalyzed using regression analysis.

2.2. I suggest to add another Tables showing the regression analysis and predictors with crude and adjusted odd ratios.

2.3. Provide confidence intervals (95%) in results for more strong interpretation.

3. Discussion: It is well written; I have some comments:

3.1. Expand the comparison with previous studies on the sample title

3.2. Support the limitations section by highlighting the limitation of low sample size.
