# Supplementary material for: Knowledge, awareness and attitude among practicing oral and maxillofacial surgeons towards tele-dentistry: A cross-sectional survey
Source: PLoS One. 2026 Apr 8;21(4):e0346591. doi: 10.1371/journal.pone.0346591 (PMC13061227; doi:10.1371/journal.pone.0346591)
Supplement: Attachment — Submitted filename: POINT BY POINT RESPONSE.pdf [file pone.0346591.s003.pdf]

POİNT-BY-POİNT RESPONSE

Manuscript Title: Knowledge, Awareness and Attitude among Practicing Oral and Maxillofacial Surgeons on Tele-Dentistry: A Cross-Sectional Survey

Manuscript Number: PONE-D-25-32213

Major Revision

Reviewer 1

Comments

First of all, thank you very much for your comments and suggestions. The comments and suggestions are valuable and very helpful for revising and improving our manuscript. In the following, we also reply to each of your comments below.

Introduction:

- please remove the repetitions .. there are lots of areas that were repeated
- Word tele-medicine.. was okay to use at the introduction. But teledentist.. is what needs to be used when mentioning dentists per se.. dont you think? ALSO.. your questionnaire refers to teledentistry. So use that world more boldly and conspicuously
- This is incorrect .. your references include several studies conducted amongst Max Facs wrt teledentistry .. recheck
- Another reason could be that this study geographical location .. differs to current study .. so the samples are different

Our response: We thank the reviewer for the valuable comments on the Introduction section. Based on these suggestions, the entire section has been thoroughly revised to improve clarity, eliminate repetitions, and ensure a more concise and consistent flow. The terminology has been corrected so that telemedicine is used only when referring to general remote healthcare, while tele-dentistry is now consistently applied when mentioning dentistry-related practices, in alignment with the focus of the study and the questionnaire. Additionally, the statement implying limited research among oral and maxillofacial surgeons has been amended.

Methods:

These are results .. exclude it from here. My understanding was that you sent the Questionnaire to 900 dental practitioners (Max Facs)

Our response: We thank the reviewer for this clarification. The statement previously written as "The study population consisted of 152 oral and maxillofacial surgeons in Türkiye." has been corrected to "The study population consisted of practicing oral and maxillofacial surgeons in Türkiye." This

modification removes the result-based interpretation and aligns the wording with the methodological description.

Inclusion criteria .. i dont think this statement is correct here

Again .. not sure what is meant having this statement here. Rethink its inclusion .You are mixing your PLAN with outcomes (or results)

Our response: We appreciate the reviewer's constructive comments on the inclusion and exclusion criteria. Based on the comments that the previous statements were unclear and seemed to mix the study plan with results, this section has been revised to present only the eligibility criteria clearly, without any interpretation or outcome-related statements. The revised version is as follows:

*"**Inclusion and Exclusion Criteria***

The inclusion criteria for this study were as follows:

- *Participants aged 23 years or older.*
- *Oral and maxillofacial surgery professionals who agreed to participate in the study (Specialist, Assistant Professor, Associate Professor, Professor).*
- *Dentists who were currently undergoing specialization or doctoral training in oral and maxillofacial surgery and who agreed to participate (Research Assistants).*

The exclusion criteria for this study were as follows:

- *Individuals from dental specialties other than oral and maxillofacial surgery.*
- *General dental practitioners.*
- *Individuals who did not provide consent to participate in the study."*

There is no description of the sample of 900 .. please include that. How did you get to that. Who helped, who you asked etc etc

Our response: We thank the reviewer for this valuable comment. Almost all actively practicing oral and maxillofacial surgeons in Türkiye are members of AÇBİD. Accordingly, in the revised Methods section, it has been clearly stated that the researchers accessed the registered email addresses of the members with the support of AÇBİD representatives (Prof. Dr. Fethi Atıl, and Prof. Dr. Ercüment Önder), and that the survey link was sent directly by the researchers. The statement added to the manuscript is as follows:

"The initial sampling frame consisted of approximately 900 members registered with AÇBİD. With the support of AÇBİD representatives, the researchers accessed only the registered email

addresses of these members; the survey link was then sent directly by the researchers to these addresses."

This is results .. not methods.

You are talking about Results etc. but then below you still detailing stats analysis

Our response: We thank the reviewer for these valuable comments. In response, the "Questionnaire Distribution" section has been revised to clearly describe only the procedural aspects of how the survey was administered, without referring to outcomes or response figures. The updated version now focuses solely on the data collection process, as follows:

"The quantitative component of the study employed convenience sampling. The Association of Oral and Maxillofacial Surgery of Türkiye (AÇBİD), a national professional organization representing oral and maxillofacial surgeons, was requested to distribute the questionnaire to its members via email. The survey remained open for a total of three months, during which six reminder emails were sent at two-week intervals to non-respondents to increase the response rate. The questionnaire was distributed to research assistants, specialist dentists, assistant professors, associate professors, and professors working in the field of oral and maxillofacial surgery. Participation was entirely anonymous, and no personally identifiable information was collected at any stage. Participants were able to revise their responses or voluntarily withdraw from the questionnaire at any point prior to submission. No incentives or compensation were provided for completing the survey."

Results:

- do not repeat info that is on table.. summarize and interpret
- Where are the numbers .. can you write clearly
- Can we replace this word .. what do you mean rises
- again .. repetition of info on Tables .. avoid this

Our response: Thank you for the reviewer's valuable comments on the Results section. Based on the feedback, this part has been revised to improve clarity, language flow, and readability, and redundant information already shown in the tables has been removed. In addition, a regression analysis was conducted as suggested by the other reviewer, and the relevant findings have been added to the Results section.

DISCUSSION MUST BE REWRITTEN please

Our response: We thank the reviewer for the valuable feedback regarding the Discussion section. In line with these comments, the section has been completely revised and rewritten to improve clarity and better reflect the study findings.

Reviewer 2:

Overall assessment

This is a valuable and timely piece of study and it does tackle an understudied area. If the study heightened the analysis and became more precise and clearer on the method the piece would be a meaningful addition to the literature on tele-dentistry use by oral and maxillofacial surgeons.

Abstract.

There some abbreviations are not identified.

The novelty (first survey among oral/maxillofacial surgeons in Türkiye) could be more prominently highlighted in the abstract. Although p-values are reported with precision, interpretability is limited because the effect sizes or the strength of associations are not stated.

1. Materials and methods

Sampling: convenience sampling method does raise some concerns regarding overall representation. The author should write how the sample size is selected? and calculated?

Questionnaire: While some parts of the study are based on previous research, there is no information on what changes were made, if any, and if their psychometric properties–validation and reliability, post-change–were determined, all these points should be considered and placed on the text.

Analysis:

Only Chi-square and Bonferroni tests are referenced. I suggest to add regression analysis, which is very important to assess the independent predictors of awareness/usage more accurately.

Clarify how the non-response rate can affect generalizability.

2. Results

2.1. The results are fair, they should cover all aspects of the study. The predictors such as age, title and independent variables can be reanalyzed using regression analysis.

2.2. I suggest to add another Tables showing the regression analysis and predictors with crude and adjusted odd ratios.

2.3. Provide confidence intervals (95%) in results for more strong interpretation.

3. Discussion:

It is well written; I have some comments:

3.1. Expand the comparison with previous studies on the sample title

3.2. Support the limitations section by highlighting the limitation of low sample size.

Comments

First of all, thank you very much for your comments and suggestions. The comments and suggestions are valuable and very helpful for revising and improving our manuscript. In the following, we also reply to each of your comments below.

1) There some abbreviations are not identified. The novelty (first survey among oral/maxillofacial surgeons in Türkiye) could be more prominently highlighted in the abstract. Although p-values are reported with precision, interpretability is limited because the effect sizes or the strength of associations are not stated.

   Our response: We thank the reviewer for the constructive comments. In line with these suggestions—and considering the additional analyses that led to revisions in the study findings—the abstract has been comprehensively rewritten.

   We thank the reviewer for the insightful comments. In response, and following the additional analyses that led to changes in the study findings, the abstract has been entirely revised. Specifically:

   All abbreviations have now been defined at first mention,

   The novelty of the study (to the best of our knowledge, the first survey among oral and maxillofacial surgeons in Türkiye) has been explicitly emphasized,

   Interpretability has been improved by adding statements on the magnitude and precision of associations, rather than relying solely on p-values.

   The revised abstract now reads as follows:

   "Tele-dentistry can be defined as the utilization of digital and telecommunication technologies to facilitate remote dental care. This study assessed the knowledge, awareness and practices related to tele-dentistry among oral and maxillofacial surgeons in Türkiye using a cross-sectional survey. A structured questionnaire was distributed to approximately 900 members of the Association of Oral and Maxillofacial Surgery of Türkiye (AÇBİD), and responses from 152 participants were analysed using descriptive statistics and binary logistic regression ($p < 0.05$). Although 46.1% of respondents reported familiarity with tele-dentistry, only 9.9% had prior experience with it. Overall awareness was moderate and attitudes toward future applications were generally positive; however, actual clinical utilization remained limited. Academic title was the only variable significantly associated with prior awareness and utilization, although the strength of this association was modest and its confidence intervals were wide. None of the predictor variables demonstrated a statistically significant association with the participants'

intention to use tele-dentistry in the future. These findings indicate that oral and maxillofacial surgeons in Türkiye are receptive to tele-dentistry but have limited practical exposure. Barriers to implementation may include insufficient training, data security concerns, diagnostic reliability issues, and lack of institutional support. Strengthening specialty training and developing system-level frameworks may support broader adoption in clinical practice. To the best of the authors' knowledge, this is the first study to examine tele-dentistry-related knowledge, awareness, and practices among oral and maxillofacial surgeons in Türkiye"

2) **Sampling:** convenience sampling method does raise some concerns regarding overall representation. The author should write how the sample size is selected? and calculated?

Our response: We thank the reviewer for these valuable comments. The missing information was added into the revised version of the Methods section (Study Design and Population) to clarify the reliability of the data as follows:

> "*The sample size was calculated using G*Power (Version 3.1.9.6; Heinrich Heine University, Düsseldorf, Germany), with a 95% confidence interval and 95% statistical power and an effect size of w = 0.411, based on parameters reported by George et al. [18]. The minimum required sample size was estimated to be 110 participants."*

[18]. George PP, Edathotty TT, Gopikrishnan S, et al. Knowledge, awareness, and attitude among practicing orthodontists on teledentistry during the COVID-19 pandemic in Kerala: a cross-sectional survey. J Pharm Bioallied Sci 2021;13(Suppl 1):846–850. doi: 10.4103/jpbs.JPBS_826_20

3) **Questionnaire:** While some parts of the study are based on previous research, there is no information on what changes were made, if any, and if their psychometric properties–validation and reliability, post-change–were determined, all these points should be considered and placed on the text.

Our response: We thank the reviewer for these valuable comments. The study was designed to provide a descriptive assessment of tele-dentistry–related knowledge, awareness, and attitudes among oral and maxillofacial surgeons, not to develop or validate a new psychometric instrument. Therefore, the questionnaire—adapted from previously published surveys [18] through the selection and combination of relevant items—was not subjected to post-modification psychometric testing (e.g., reliability or validity analyses). This methodological consideration has now been explicitly clarified in the revised manuscript in the *Limitations* section, as follows:

> "Furthermore, *the questionnaire used in this study was adapted from previously published surveys by selecting and combining relevant items. Since it was not subjected*

to full post-modification psychometric validation (e.g., reliability or validity testing), the measurement precision may be limited."

4) **Analysis:** Only Chi-square and Bonferroni tests are referenced. I suggest to add regression analysis, which is very important to assess the independent predictors of awareness/usage more accurately.

   Our response: We appreciate the reviewer's valuable comment. We agree that regression analysis is important for accurately identifying the independent predictors of tele-dentistry awareness and usage. In addition to Pearson's Chi-Square test and Bonferroni correction applied for multiple comparisons, binary logistic regression analysis was performed to determine the variables independently associated with (1) having heard of tele-dentistry, (2) prior use of tele-dentistry, and (3) intention to use tele-dentistry in the future. Odds ratios (OR) with 95% confidence intervals were calculated for each model.

   This information has been clarified in the Methods section (Statistical Analysis and Study Variables), and the corresponding results are presented in Tables 4, 5, and 6, which report the regression outcomes.

5) Clarify how the non-response rate can affect generalizability.

   Our response: We thank the reviewer for these valuable comments. We acknowledge that the non-response rate may influence the generalizability of the findings. As participation was based on voluntary responses to an online questionnaire distributed via AÇBİD, individuals with a greater interest in tele-dentistry may have been more likely to participate, potentially introducing selection bias. Additionally, since the number of surgeons who received or viewed the questionnaire link but did not respond could not be determined, the extent of non-response bias could not be formally evaluated.

   This methodological limitation has now been clearly addressed in the Limitations section of the revised manuscript as follows:

   "Second, participant recruitment was based on voluntary responses to a Google Forms link distributed through AÇBİD and the non-response rate for this study was could not be determined. This may have introduced selection bias, as individuals with a greater interest in tele-dentistry may have been more inclined to participate. Furthermore, because the number of surgeons who received or viewed the link but did not to respond was unknown, the extent of non-response bias could not be evaluated.

As a result, the generalizability of the findings to all OMFS surgeons may be limited. Nonetheless, participation from multiple institutions may have partially mitigated these concerns."

6) **(2) Results**

(2.1.) The results are fair, they should cover all aspects of the study. The predictors such as age, title and independent variables can be reanalyzed using regression analysis.

(2.2.) I suggest to add another Tables showing the regression analysis and predictors with crude and adjusted odd ratios.

(2.3.) Provide confidence intervals (95%) in results for more strong interpretation.

Our response We thank the reviewer for these constructive observations regarding the presentation of the results.

(2.1) In line with the suggestion, the results section has been expanded to cover all relevant aspects of the study. Independent predictors including age, academic title, and gender were reanalyzed using binary logistic regression.

(2.2) Additional tables (Tables 4, 5, and 6) have been included to present the regression analysis outcomes, showing both crude and adjusted odds ratios for predictors of (1) awareness of tele-dentistry, (2) prior use of tele-dentistry, and (3) intention to use tele-dentistry in the future.

(2.3) Confidence intervals (95% CI) have been added alongside odds ratios to strengthen the interpretation of the regression results.

Tables 4,5, and 6 have been added to the revised result section.

Table 4. Binary logistic regression analysis of independent variables affecting the likelihood of hearing about tele-dentistry.

	Have you heard of tele-dentistry?		Univariate		Multiple	
	No N (%)	Yes N (%)	OR (%95 CI)	p-value	OR (%95 CI)	p-value
**Age**						
23-35	66 (61.1)	42 (38.9)	Reference			
35-45	8 (26.7)	22 (73.3)	4.32 (1.76 – 10.6)	**0.001**	1.93 (0.54 – 6.89)	0.309
45>	2 (25)	6 (75)	4.71 (0.91 – 24.46)	0.065	1.05 (0.07 – 16.08)	0.975
**Gender**						
Male	48 (53.9)	41 (46.1)	Reference			
Female	28 (49.1)	29 (50.9)	1.21 (0.62 – 2.36)	0.571	1.47 (0.7 – 3.07)	0.307
**Title**						
Research Assistant	46 (67.6)	22 (32.4)	Reference			
Specialist	17 (58.6)	12 (41.4)	1.48 (0.6 – 3.62)	0.395	1.36 (0.53 – 3.5)	0.526
Assistant Professor	6 (30)	14 (70)	4.88 (1.65 – 14.41)	**0.004**	3.96 (1.27 – 12.34)	**0.018**
Associate Professor	5 (27.8)	13 (72.2)	5.44 (1.72 – 17.17)	**0.004**	3.38 (0.72 – 15.88)	0.122
Professor	2 (18.2)	9 (81.8)	9.41 (1.87 – 47.27)	**0.006**	8.16 (0.57 – 116.65)	0.122

Cox&Snell R^2=%14.6; Nagelkerke R^2=%19.5; Frequency (percentage); OR: Odds Ratio. CI: Confidence Interval
Bold values indicate statistical significance ($p < 0.05$).

Table 5. Binary logistic regression analysis of independent variables with previous use of tele-dentistry

	Have you used tele-dentistry before?		Univariate		Multiple	
	No N (%)	Yes N (%)	OR (%95 CI)	p-value	OR (%95 CI)	p-value
**Age**						
23-35	103 (92)	9 (8)	Reference			
35-45	27 (90)	3 (10)	1.27 (0.32- 5.02)	0.732	0.11 (0.01 – 1.98)	0.135
45>	5 (62.5)	3 (37.5)	6.87 (1.41- 33.51)	**0.017**	0.08 (0 – 3.45)	0.191
**Gender**						
Male	80 (88.9)	10 (11.1)	Reference			
Female	55 (91.7)	5 (8.3)	0.73 (0.24 – 2.25)	0.580	1.06 (0.3 – 3.81)	0.926
**Title**						
Research Assistant	65 (92.9)	5 (7.1)	Reference			
Specialist	29 (93.5)	2 (6.5)	0.9 (0.16 – 4.89)	0.900	1.1 (0.2 – 6.07)	0.913
Assistant Professor	19 (95)	1 (5)	0.68 (0.08 – 6.22)	0.736	0.94 (0.1 – 8.77)	0.959
Associate Professor	16 (88.9)	2 (11.1)	1.63 (0.29 – 9.15)	0.582	7.46 (0.68 – 82.28)	0.101
Professor	6 (54.5)	5 (45.5)	10.83 (2.43 – 48.32)	**0.002**	119.57 (3.46 – 4136.53)	**0.008**

Cox&Snell R^2=%8.8; Nagelkerke R^2=%18.5; Frequency (percentage); OR: Odds Ratio. CI: Confidence Interval
Bold values indicate statistical significance ($p < 0.05$).

Table 6. Binary logistic regression analysis of independent variables affecting the likelihood of considering the use of tele-dentistry in the future

	In the future, will you practice tele-dentistry?		Univariate		Multiple	
	No N (%)	Yes N (%)	OR (%95 CI)	p-value	OR (%95 CI)	p-value
**Age**						
23-35	12 (20)	48 (80)	Reference			
35-45	2 (8)	23 (92)	2.88 (0.59 – 13.92)	0.189	---	---
45>	4 (50)	4 (50)	0.25 (0.05 – 1.15)	0.075	---	---
**Gender**						
Male	14 (22.2)	49 (77.8)	Reference			
Female	4 (13.3)	26 (86.7)	1.86 (0.56 – 6.22)	0.315	1.73 (0.49 – 6.09)	0.394
**Title**						
Research Assistant	6 (17.6)	28 (82.4)	Reference			
Specialist	7 (36.8)	12 (63.2)	0.37 (0.1 – 1.33)	0.126	0.39 (0.11 – 1.41)	0.150
Assistant Professor	1 (8.3)	11 (91.7)	2.36 (0.25 – 21.9)	0.451	2.33 (0.25 – 21.78)	0.458
Associate Professor	1 (5.6)	17 (94.4)	3.64 (0.4 – 32.91)	0.250	3.87 (0.42 – 35.22)	0.230
Professor	3 (30)	7 (70)	0.5 (0.1 – 2.51)	0.400	0.55 (0.11 – 2.79)	0.467

Cox&Snell R^2=%8.9; Nagelkerke R^2=%14.2; Frequency (percentage); OR: Odds Ratio. CI: Confidence Interval
Bold values indicate statistical significance ($p < 0.05$).

7) **Discussion**: It is well written; I have some comments:

3.1. Expand the comparison with previous studies on the sample title

Our response: We thank the reviewer for your valuable comment. The Discussion section has been fully rewritten in accordance with your recommendations.

8) Support the limitations section by highlighting the limitation of low sample size

Our response: We thank the reviewer for this valuable comment. In response, the limitations section has been revised to emphasize the impact of the sample size. Although the sample size was statistically adequate, it remains relatively limited and may influence the generalizability of the findings. The following sentence has been added to clarify this point.

"Although the sample size was statistically adequate (n = 152) and comparable to previous surveys [18], the findings may not be fully generalizable to all OMFS surgeons in Türkiye."
